# Barriers to effective management of type 2 diabetes mellitus in primary healthcare facilities

**DOI:** 10.4102/jphia.v16i1.1420

**Published:** 2025-09-10

**Authors:** Ntlogeleng M. Mogale, Thembelihle S. Ntuli, Paul K. Chelule

**Affiliations:** 1 Department of Public Health, Faculty of Health Care Sciences, Sefako Makgatho Health Sciences University, Pretoria, South Africa; 2 Department of Statistical Sciences, Faculty of Science and Technology, Sefako Makgatho Health Sciences University, Pretoria, South Africa

**Keywords:** type 2 diabetes mellitus, primary healthcare, barriers, workload pressure, effective management

## Abstract

**Background:**

In 2024, type 2 diabetes mellitus (T2DM) – a public health challenge – affected 589 million adults worldwide. In South Africa, the prevalence is estimated at 15%, contributing to approximately 3.4 million deaths. Achieving optimal glycaemic control in patients is challenging, resulting in preventable complications and deaths. Systemic reforms and targeted interventions are urgently required.

**Aim:**

To identify barriers faced by healthcare professionals (HCPs) to effectively manage patients with T2DM.

**Setting:**

A survey was conducted in the Tshwane Metropolitan Municipality, Gauteng province, South Africa.

**Methods:**

The study involved 205 HCPs across 22 clinics and 6 community health centres from May 2022 to June 2022. Data were analysed descriptively. Categorical variables were compared using Fisher’s exact test and a *p*-value of < 0.05 was considered significant.

**Results:**

Most participants were nurses (84%), < 50 years (65%), female (87%), black people (64.7%), and > 10 years experience (65%) and had academic and in-house training on T2DM (38.5%). Key barriers faced by HCPs in managing patients with T2DM included workload making it difficult monitor patients (53%) and screen for complications (57%), time pressures to deliver quality care (59%) and educate patients (69%), inadequate insulin initiation knowledge (68%) and lack of continuity of care (62%).

**Conclusion:**

Excessive workloads, insufficient staffing, time pressures, insulin inertia, and knowledge gaps – impede the delivery of personalised care, patient education and monitoring.

**Contribution:**

Addressing these challenges will require collaborative care models, workforce optimisation, targeted training, better resource allocation and health technology and can significantly improve patient outcomes and alleviate burden among HCPs.

## Introduction

Globally, type 2 diabetes mellitus (T2DM) is a growing public health concern that has become increasingly prevalent in recent years. In 2024, it is estimated that 589 million adults aged between 20 and 79 years were living with diabetes, worldwide, and this number is projected to reach 853 million by 2050.^1^ In the African continent, the situation is particularly worrisome, with projections indicating a significant rise in the prevalence of T2DM from 3.2% to 3.7% by the year 2040.^2^ This troubling trend is also reflected in South Africa, where studies have reported a wide range of prevalence rates, from 7% to as high as 15% among the adult population.^2,3,4,5,6^ In another study, the pooled prevalence of T2DM in South Africa was estimated at 15.25%.^7^ In 2024, approximately 3.4 million adults aged 20–79 years died because of diabetes or related complications and the proportion of deaths related to diabetes in the same age group in South Africa, is between 5% and 8%.^1^

The prevention of T2DM-related complications, including retinopathy, nephropathy, neuropathy, cardiovascular disease, amputation and premature death, depends on early and efficient glycaemic control among patients.^2,8,9^ Maintaining optimal glycaemic control, as measured by haemoglobin A1c (HbA1c) levels below 7%, continues to be a significant challenge for many individuals living with T2DM. Despite advances in diabetes management strategies and treatments, a substantial number of patients struggle to consistently achieve these recommended glycaemic targets. Studies conducted in hospitals and primary care settings show that individuals with T2DM are not adequately managed; glycaemic target attainment rates or the proportion of T2DM patients whose glycated HbA1c is successfully lowered to less than or equal to 7%, range from 10% to 35%.^10,11,12,13,14,15^

Research indicates that patients with T2DM encounter numerous systemic barriers to effective glycaemic control, including poor adherence to medication and lifestyle regimens, socioeconomic constraints, irregular blood glucose monitoring, limited access to healthcare resources and education, staff shortages resulting in excessive workloads and clinicians’ hesitancy to escalate treatment when clinically indicated.^16,17,18,19,20^ These compounding challenges highlight the urgent need for comprehensive health system reforms to improve diabetes care delivery and outcomes. The recent enactment of South Africa’s National Health Insurance (NHI) Bill represents a pivotal step towards universal health coverage, building upon earlier initiatives including the 2011 NHI Green Paper, 2012 pilot projects and 2015 White Paper. However, full NHI implementation remains a gradual process requiring multisectoral collaboration.^21^

South Africa’s health system strengthening approach, aligned with World Health Organization (WHO) building blocks (human resources, financing, governance, information systems, medical products and service delivery), is being operationalised through NHI and primary healthcare re-engineering. The phased NHI implementation commenced with quality improvement initiatives (2012–2016), including facility audits and establishment of the Office of Health Standards Compliance. The subsequent phase (2017–2021) focused on legislative development and creating NHI fund structures, while the current phase (2022–2025) aims to achieve full NHI functionality.^21,22^ These reforms are particularly crucial for chronic disease management, where systemic weaknesses most acutely affect patient outcomes.

To inform targeted interventions that enhance both patient outcomes and healthcare worker support, this study aimed to examine the challenges and barriers faced by healthcare professionals in managing and monitoring patients living with T2DM within Tshwane District Municipality. By elucidating frontline barriers, from resource limitations to workflow inefficiencies, the research aims to provides evidence to strengthen diabetes care within the evolving NHI framework, ultimately contributing to more responsive chronic disease management systems under universal health coverage.

## Research methods and design

### Study design

This cross-sectional descriptive study was conducted among healthcare professionals employed in clinics and community health centres across the Tshwane Metropolitan Municipality, in the Gauteng province of South Africa. The study took place between May 2022 and July 2022.

### Study setting

The Tshwane Metropolitan Municipality, which is one of the three metropolitan municipalities in the Gauteng province of South Africa, is the administrative capital of the country. This municipality is divided into 7 regions and encompasses a total of 103 clinics, 8 community health centres (CHCs), 1 regional hospital, 2 academic hospitals and 4 district hospitals. The study was conducted across 5 randomly selected regions and included a sample of 22 clinics and 6 CHCs.

### Study population, inclusion and exclusion criteria

The study population comprised nurses and medical doctors working in the PHC clinics and CHCs. Participants were above 18 years of age, with at least 1 year of working experience in providing comprehensive treatment, care and management for patients diagnosed with T2DM. Healthcare professionals working in hospitals or private practice, those with limited experience in treating patients with T2DM, those unwilling or unable to provide informed consent or absent at the time of data collection were excluded from the study.

### Sample size and sampling strategies

The actual numbers of HCP who treated patients with T2DM was unknown and differed per facility, ranging from 2 to 20. Therefore, an estimated 2084 HCPs in Tshwane was used to determine the required minimum sample size (personal communication). The Cochrane’s sample size calculation for proportions was used to calculate the minimum sample size using a distribution rate of 80%, 5% margin of error and 95% confidence interval. The minimum required sample size was 246, and this was increased to 250.

All available HCPs responsible for the care and treatment of patients with T2DM and had agreed to participate, where included in the study. Participants were recruited as a group in their respective facilities, mostly during their morning meetings. The purpose of the study and rights to voluntary participation were presented prior to obtaining verbal informed consent.

### Data collection

A structured, standardised and self-administered questionnaire was used to collect data from the study participants. Literature was utilised to guide the development of the main tool.^20,23^ The original tool had 5 main sections, namely the sociodemographic information, management and monitoring of patients, barriers to effective management of patients and behavioural intention of healthcare providers to use real-time continuous glucose monitoring among patients with T2DM. The tool had closed and a few open-ended questions. The tool had 20 questions that examined barriers to effective management of patients with T2DM. Of those, 12 focused on individual-level barriers, while 6 addressed system-level barriers and the rest focused on sociodemographic characteristics. The individual-level barrier questions explored issues such as healthcare providers’ knowledge, skills, workload, time constraints, language barriers and perceived roles in the treatment and care of patients. The system-level barrier questions investigated resource and practice-related challenges.

Participants were required to indicate whether they agreed or disagreed with the statements. An interview lasted for about 10 min to 20 min. Data collection lasted for about 10 weeks, from May 2022 to July 2022. Google Forms (Google, LLC) was used to capture data to minimise data entry errors. After the collection period, data were exported into Microsoft Excel (Microsoft Corp) prior to importation into STATA for cleaning, coding and analysis.

### Data analysis

Data analyses were conducted using STATA 17 SE (College Station, Texas: StataCorp, LLC, United States). Descriptive statistics were presented as mean and standard deviation for continuous variables and as count and proportion for categorical variables. The researchers employed Fisher’s exact test to compare the perceptions of nurses and doctors regarding the barriers encountered in providing care for patients with T2DM. A *p*-value of less than or equal to 0.05 was considered to be statistically significant for the purposes of this analysis.

### Ethical considerations

Ethical clearance was obtained from Sefako Makgatho Health Sciences University Research Ethics Committee (No. SMUREC/H/318/2021:PG).

Permission to conduct the study was obtained from the Gauteng Provincial Department of Health and the Tshwane District Research Committee. Further permission was obtained from the facility operations managers of the primary healthcare facilities that participated in the study. This study was conducted in line with the Declaration of Helsinki. Participants were informed about the purpose of the study, their rights to voluntary participation and withdrawal from the study at any point, without any consequences. Participants preferred to consent verbally and this was obtained prior to completing the questionnaires. The study was anonymised to ensure confidentially and privacy of the participants. All identifiable information of the facilities were de-linked.

## Results

### Demographic characteristics

A total of 250 questionnaires were distributed, and of those, 205 were completed, which resulted in a response rate of 82%. The mean age of the participants was 44 ± 11.1 years (ranged from 24 years to 67 years). Most participants were over 40 years old (62%, *n* = 128), female (87%, *n* = 179) and of black African descent (86%, *n* = 177). The majority were nurses (84%, *n* = 173), in contrast with medical doctors (16%, *n* = 32) (Table 1). Although not included in the table, of the nurses and doctors in the study, 91% (*n* = 157) were professional nurses while 47% (*n* = 15) were medical officers and registrars, respectively. Most of the participants were employed in the primary healthcare clinics (81%, *n* = 165) and 65% (*n* = 132) had more than 10 years of working experience in the setting. In terms of clinical experience of managing patients with T2DM, 35% (*n* = 72) had more than 10 years of experience. About 39% (*n* = 79) reported to have received training on T2DM while pursuing their degrees and during in-house training workshops.

**TABLE 1:** Demographic information of the participants, (*N* = 205).

Variables	Frequency (*n*)	%
**Age (years)**		
> 30	29	14.1
30–39	48	23.4
40–49	56	27.3
50–59	57	27.8
60+	15	7.3
**Sex**		
Female	179	87.3
Male	26	12.7
**Ethnicity**		
Black people	177	86.3
Other racial groups	28	13.7
**Profession**		
Nurses	173	84.4
Doctors	32	15.6
**Facility type**		
Primary healthcare clinics	165	80.5
Community health centres	40	19.5
**Years of experience as a healthcare professional**		
< 5	46	22.4
5–10	26	12.7
> 10	132	64.4
Missing	1	0.5
**Years of experience treating patients with T2DM**		
< 5	62	30.2
5–10	58	28.3
> 10	72	35.1
Missing	13	6.3
**Type of training received on T2DM**		
Academic	65	31.7
In-service and academic	79	38.5
Workshops and academic	37	18.0
Workshops, in-service and academic	20	9.8
Missing	4	2.0

Note: Other racial groups: white people, Indian people and coloured (mixed race) people. Doctors: family physicians, medical officers, interns and registrars; Nurses: Professional, staff, assistant, community seervice and student nurses: staff, assistant, community service and student nurses.

T2DM, type 2 diabetes mellitus.

### Barriers to effective management of patients with type 2 diabetes mellitus

Table 2 details the healthcare professionals’ perspectives on the key obstacles and challenges they face in the effective management of patients with T2DM.

**TABLE 2:** Barriers to effective management of patients with type 2 diabetes mellitus.

Barriers	Statements	All				Nurses				Doctors				*p*-value
		Agree		Disagree		Agree		Disagree		Agree		Disagree		
		*n*	%	*n*	%	*n*	%	*n*	%	*n*	%	*n*	%
Workload	The workload makes it very difficult for me to effectively monitor patients with diabetes mellitus.	108	53	97	47	84	49	89	51	24	75	8	25	0.007
	In most cases, I’m unable to screen patients for complications related to diabetes due to other work-related pressures.	117	57	88	43	92	53	81	47	25	78	7	22	0.011
Time pressure	Unable to deliver quality care to patients with diabetes mellitus due to time pressures.	120	59	85	41	94	54	79	46	26	81	6	19	0.006
	I always have enough time to sit with patients and educate them about the disease (diabetes) and lifestyle modification.	64	31	141	69	58	34	115	64	6	19	26	81	0.145
Language barriers	The language barrier is a challenge to offering proper care to patients with diabetes mellitus.	65	32	140	68	51	29	122	71	14	44	18	56	0.147
Perceived role	It is my role to screen for diabetes complications in the patients I am seeing.	181	88	24	12	149	86	24	14	32	100	0	0	0.017
Knowledge and skills	I have enough knowledge and skills to intensify diabetes treatment.	166	81	39	19	135	78	38	22	31	97	1	3	0.012
	I have been sufficiently trained to provide health-related lifestyle modification advice to patients.	167	81	38	19	138	80	35	20	29	91	3	9	0.215
	I lack adequate knowledge and skills to know whether patients are controlled or not.	33	16	172	84	29	17	144	83	4	13	28	87	0.793
	I would sometimes delay treatment because of fear that the patient might experience side effects from a certain medication.	28	14	177	84	21	12	152	88	7	22	25	78	0.161
	I have enough knowledge and skills to initiate insulin therapy for patients who required it.	65	32	140	68	38	22	135	78	27	84	5	16	< 0.001
	I have enough knowledge and skills to make recommendations for treatment intensification and/or modification.	149	73	56	27	121	70	52	30	28	87	4	13	0.051
Resources and monitoring of glycaemic control	The facility lacks adequate equipment to effectively manage and monitor patients with diabetes.	58	28	147	72	47	27	126	73	11	34	21	66	0.401
	In my facility, some protocols enable me to structure diabetes care.	157	77	48	23	135	78	38	22	22	69	10	31	0.262
	Access to HbA1c testing is a serious challenge in our facility and as such, it is not done as it should be.	24	12	181	88	23	13	150	87	1	3	31	91	0.136
	My facility has a system that allows me to be able to know what was done to the patient in their previous visit.	182	89	23	11	152	88	21	12	30	94	2	6	0.541
	In my facility, we rely on the patient’s file to know what was done to them in their previous visit.	146	71	59	29	124	72	49	28	22	69	10	31	0.832
Practices	The lack of continuity of care for patients makes it difficult for me to monitor patients with diabetes, effectively.	128	62	77	38	104	60	69	40	24	75	8	25	0.118
	In my setting, the coordination of diabetes care is often a challenge.	52	25	153	75	42	24	131	76	10	31	22	69	0.387

#### Workload

Overall, more than half of the participants indicated that heavy workloads imposed a challenge in their ability to effectively manage and monitor patients with diabetes mellitus (53%, *n* = 108), and adequately screen those with complications related to the disease (57%, *n* = 117). Compared to nurses, doctors, significantly reported to have heavy workload and demanding schedules making it extremely challenging for them to effectively monitor patients (75% vs. 49%, *p* = 0.007) and thoroughly screen patients with T2DM for any related complications (78% vs. 53%, *p* = 0.011).

#### Time pressure

Many of the participants (59%, *n* = 120) reported that the time pressure they face in their clinical practice makes it challenging for them to consistently deliver high-quality care to patients with diabetes. A significant difference was observed between nurses and doctors and this issue was notably more prevalent among doctors than nurses (81% vs. 54%, *p* = 0.006). Because of the time constraint, 69% (*n* = 141) of the participants lacked the time to sit with patients and comprehensively educate them about the disease and lifestyle modification. Although most doctors reported not having adequate time than nurses (81% vs. 64%, *p* = 0.145), the results were, however, not statistically significant.

#### Language barriers

Several participants (32%, *n* = 65) reported that language barrier presents a challenge in providing appropriate care for patients with T2DM. Doctors were more likely to experience language barriers compared to nurses (44% vs. 29%, *p* = 0.147), although the results were not statistically significant.

#### Perceived role

Most participants (88%, *n* = 181) acknowledged that it is their responsibility to screen patients for complications related to diabetes. A statistically significant difference was observed between doctors and nurses (100% vs. 86%, *p* = 0.017).

#### Knowledge and skills

Most (81%, *n* = 166) of the participants indicate that they possessed the necessary knowledge and skills to intensify diabetes treatment, and this was significantly reported by doctors compared with nurses (97% vs. 78%, *p* = 0.012). The results further indicate that most participants (81%, *n* = 167) were adequately trained to provide patients with health-related lifestyle modification guidance, with doctors more often making this claim than nurses, even though the difference was not statistically significant (91% vs. 80%, *p* = 0.215). A small proportion (16%, *n* = 33) of participants indicated that they lacked the necessary knowledge and skills to determine whether patients were properly managed, and this was more frequently reported by nurses than doctors; however, this difference was also not statistically significant (17% vs. 13%, *p* = 0.793).

Most participants (84%, *n* = 177) disagreed with the idea that they might occasionally delay treatment for patients because of concerns about potential medication side effects. There was no significant difference observed between nurses and doctors (88% vs. 78%, *p* = 0.161). Several participants (32%, *n* = 65) reported having adequate knowledge and skills to commence insulin treatment for patients who require it, with a notably larger proportion of doctors possessing this knowledge compared to nurses (84% vs. 22%, *p* = 0.001). About 73% (*n* = 149) also reported that they have sufficient knowledge and skills to recommend treatment intensification or adjustment, with no significant disparity observed between doctors and nurses (69% vs. 72%, *p* > 0.05)

#### Resources and monitoring of glycaemic control

The majority of the participants reported that the facility has appropriate equipment to effectively manage and monitor patients with diabetes (72%, *n* = 147), has some protocols in place to structured care related to diabetes (77%, *n* = 157), has access to HbA1c testing, which is not a significant issue (88%, *n* = 181), has a system that allows clinicians to be aware of the patient’s previous visit details (89%, *n* = 182) and relies on the patient’s file to track their history (71%, *n* = 146). In addition, there was no notable difference between doctors and nurses in these aspects (*p* > 0.05).

#### Practices related to diabetes care

A small number of participants (25%, *n* = 52) reported that coordinating diabetes care is a challenge in their practice setting, with no difference observed between doctors and nurses (31% vs. 24%, *p* = 0.387). However, many (62%, *n* = 128) stated that the lack of continuity of care for patients makes it hard for them to effectively monitor and manage patients with T2DM, and again no significant difference between doctors and nurses was observed (75% vs. 60%, *p* = 0.118).

## Discussion

The study aimed to investigate the key barriers hindering effective management of patients with T2DM. Our findings revealed that excessive workloads posed significant challenges for healthcare professionals, particularly in monitoring patients and screening for diabetes-related complications. Notably, this issue was more frequently reported by doctors compared to nurses. Research has consistently shown that inadequate staffing results in elevated workloads, creating a high patient-to-clinician ratio.^19,20^ Such conditions often lead to diminished quality of care, increased medical errors, lower patient satisfaction and burnout among healthcare providers.^24,25,26^ Addressing this issue requires strategic interventions, such as reassessing staffing levels, enhancing workflow efficiency and leveraging technological solutions. Implementing these changes could help alleviate the strain on healthcare teams and foster more effective and holistic diabetes management.

Another challenge faced by healthcare professionals in the management of T2DM is the significant work-related pressure affecting the quality of care and time to educate patients about the disease and lifestyle modification. Several studies have reported similar constraints faced by healthcare professionals,^19,20^ which may contribute to difficulty in providing comprehensive and personalised care for patients with diabetes.^20,27,28^ These pressures can potentially limit the time and attention healthcare professionals dedicate to educating patients, monitoring their condition and implementing effective treatment plans.^18,29^ Investing in health technology, such as conversational agents (tailored to patients’ literacy, age, culture and socioeconomic status), continuous glucose monitoring and electronic health records can help address barriers in patient education, monitoring and management. These tools have the potential to empower patients to manage their conditions while reducing the strain on healthcare professionals.^30,31,32^

In this study, few of the participants had enough knowledge and skills to initiate insulin therapy for patients who required it, and doctors were more likely to be knowledgeable than nurses in this regard. This is consistent with another study where most healthcare professionals reported lacking confidence in their ability to initiate insulin therapy citing insufficient information or resources.^33^ Developing and maintaining up-to-date knowledge and skills in the comprehensive management of patients, including the initiation and titration of insulin therapy, is critical for providing high-quality, evidence-based care.^27^ Therefore, nurses need to be continuously educated about the proper protocols and best practices for managing insulin therapy for the patients in order to deliver the most effective care and improved outcomes.^34^

A few participants also indicated that the persistent unavailability of essential resources remained a significant challenge for the effective management of patients with T2DM. Interestingly, this issue was more frequently reported by doctors than nurses, although the difference in their responses was not statistically significant. The unavailability of essential resources, such as equipment and supplies, needs to be addressed urgently in order to ensure the provision of high-quality diabetes care. A multicentric cross-sectional study conducted in the Democratic Republic of the Congo health facilities found that most of the facilities lack of equipment for management of patients living with diabetes.^35^ Studies conducted in the Tshwane reported that some of the critical equipment required to provide high-quality diabetes-related care were unavailable to the healthcare professionals.^23,36^

The findings of this study suggest that the majority of participants reported difficulties in effectively monitoring patients with T2DM because of a lack of continuity of care. This concern was more prevalent among physicians compared to nurses although the differences were not statistically significant. Continuing medical care, patient self-management education and support can help prevent acute complications and reduce the risk of long-term T2DM complications.^37^ Bosire and colleagues describe continuity of care as collaborative care, which they often find lacking in primary healthcare facilities because of insufficient staffing.^28^ The authors suggest that collaborative care can facilitate patient-centred care, particularly when provided by a team. Similarly, Haque and colleagues found that inadequate staffing contributes to a lack of continuity of care, which represents a significant barrier.^38^ Therefore, it is crucial to implement strategies that ensure ongoing and coordinated care for patients, which would ultimately enhance disease management and patient outcomes.

### Limitations

There are several limitations to be noticed in this study. Because of the small number of healthcare professionals dedicated to the treatment and care of patients with T2DM, this study used consecutive sampling technique and recruited eligible participants who were available at the facility on the day of data collection, thus potentially introducing self-selection bias. Although the study took place in 22 clinics and 6 CHCs, the small sample size may result in some potential external generalisability of the findings. Furthermore, the study relied on self-reported data from the healthcare professionals, which may have aspects of social desirability bias, leading to favourable responses in other aspects. A review of clinical records and audits could provide more accurate insights into diabetes care practices because these may vary across clinics and CHCs. The availability of resources, clinic size, patient load and infrastructure were not accounted for in this study, and this could have had an influence on the overall responses related to care provision. Despite these limitations, this study has shed some light on the management and care of patients with T2DM and highlighted key barriers that are also supported by the literature and further warrants attention.

## Conclusion

This study identifies several key barriers to effective T2DM management, including excessive workloads among healthcare professionals, inadequate staffing leading to high patient-clinician ratios and time constraints that compromise personalised care and patient education. Knowledge gaps in insulin therapy initiation (especially among nurses) and persistent shortages of essential medical resources further hinder optimal care delivery. The lack of continuity of care, exacerbated by insufficient staffing, emerges as a critical concern, aligning with the existing literature on collaborative care models. These findings underscore the need for comprehensive interventions including workforce optimisation, targeted training programmes, improved resource allocation and enhanced care coordination and use of health technology to address both healthcare professionals’ challenges and patient outcomes in T2DM management.
